# A Consistency-Based Feature Selection Method Allied with Linear SVMs for HIV-1 Protease Cleavage Site Prediction

**DOI:** 10.1371/journal.pone.0063145

**Published:** 2013-08-23

**Authors:** Orkun Öztürk, Alper Aksaç, Abdallah Elsheikh, Tansel Özyer, Reda Alhajj

**Affiliations:** 1 eSNAg Research Group, Department of Computer Engineering, TOBB University, Ankara, Turkey; 2 Department of Computer Science, University of Calgary, Calgary, Alberta, Canada; 3 Department of Computer Science, Global University, Beirut, Lebanon; 4 Raccoon Software Computer R&D Ltd., Ankara, Turkey; National Institute of Infectious Diseases, Japan

## Abstract

**Background:**

Predicting type-1 Human Immunodeficiency Virus (HIV-1) protease cleavage site in protein molecules and determining its specificity is an important task which has attracted considerable attention in the research community. Achievements in this area are expected to result in effective drug design (especially for HIV-1 protease inhibitors) against this life-threatening virus. However, some drawbacks (like the shortage of the available training data and the high dimensionality of the feature space) turn this task into a difficult classification problem. Thus, various machine learning techniques, and specifically several classification methods have been proposed in order to increase the accuracy of the classification model. In addition, for several classification problems, which are characterized by having few samples and many features, selecting the most relevant features is a major factor for increasing classification accuracy.

**Results:**

We propose for HIV-1 data a consistency-based feature selection approach in conjunction with recursive feature elimination of support vector machines (SVMs). We used various classifiers for evaluating the results obtained from the feature selection process. We further demonstrated the effectiveness of our proposed method by comparing it with a state-of-the-art feature selection method applied on HIV-1 data, and we evaluated the reported results based on attributes which have been selected from different combinations.

**Conclusion:**

Applying feature selection on training data before realizing the classification task seems to be a reasonable data-mining process when working with types of data similar to HIV-1. On HIV-1 data, some feature selection or extraction operations in conjunction with different classifiers have been tested and noteworthy outcomes have been reported. These facts motivate for the work presented in this paper.

**Software availability:**

The software is available at http://ozyer.etu.edu.tr/c-fs-svm.rar.

The software can be downloaded at esnag.etu.edu.tr/software/hiv_cleavage_site_prediction.rar; you will find a readme file which explains how to set the software in order to work.

## Background

Acquired immune deficiency syndrome (AIDS) is a pandemic caused by HIV. AIDS is one of the major diseases seriously threatening lives of people in many parts of the world. According to 2009 data released by the World Health Organization (WHO), 33.4 million people around the world suffer from AIDS [1]. Despite the intense efforts of the health organizations, no cure has been discovered and reported effective yet, except the treatments that inhibit the growth of the disease. In order to prevent the spread of the virus within the body and to reduce death cases from AIDS, HIV-1 protease inhibitors are developed.

HIV-1 protease is an enzyme that requisites the life-cycle of HIV which cleaves protein to its component peptides [2], [32], [40]. Since HIV-1 protease is essential for the replication of the virus, the conducted research has concentrated mostly on preventing the chemical action of protease by binding molecules formed through HIV-1 protease inhibitor drugs to their active site. The mission of inhibitors is to occupy the active site of HIV-1 protease with the purpose of prohibiting its normal functionality [3], [4]. Unfortunately, this is a fairly difficult process as there is no certainty of a discovered pattern on the cleavage sites of enzymes.

Protease-peptide interaction often resembles the “lock and key” model, where a sequence of amino acids fit as a key to the active site in the protease [5]. For the HIV-1 protease case, it is known that an octapeptide region of protein composes susceptible sites whose amino acid residues are sequentially symbolized by 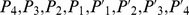, and their corresponding parts in the protease are denoted 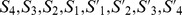, respectively. There are rare situations where some proteins include one subsite less or more (heptapeptide or nonapeptide) [6]. However, the dataset used in our work does not contain any heptamer or nonamer sequences, hereby no preprocessing is performed for any instance to obtain octamer sequences. The crucial point here is determining which octamers can or cannot be cleaved by the HIV-1 protease while searching for potential inhibitors. Nevertheless, by considering the existence of 20 amino acids, 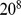 possible combinations of sequences can be mentioned. It would be very challenging to test 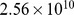 octapeptides in a laboratory environment to discriminate cleaved from uncleaved instances. For this purpose, as part of the effort to develop effective and feasible techniques to tackle the problem, accurate and robust computational methods have been implemented and tested to speed up the prediction process [7], [8].

From computational viewpoint, the problem described above can be seen as a binary classification task where an input sequence is required to be assigned a label, either cleavable or uncleavable. Several machine learning based techniques, mainly based on the classification task, have been proposed for handling the HIV-1 protease cleavage site prediction problem. These techniques utilize Neural Networks [9], Support Vector Machines (SVMs) [10], and Markov models [11].

In the work described in this paper, we developed a new approach to deal with the HIV-1 protease cleavage site prediction problem. We have primarily concentrated on the feature selection process (rather than the classification issue). This can be seen as an important step before or within the classification task; it has also been investigated in [12]–[14] specifically for the HIV-1 problem. Furthermore, interested readers can refer to the works described in [15], [16] for a review and for more information about the HIV-1 cleavage site prediction problem.

Feature selection techniques are mainly divided into three categories: Filter, Wrapper, and Embedded methods. Filter based methods assess how relevant the feature is by looking at its intrinsic properties. In most cases features are ranked according to the relevance score. Feature subset is selected in a pre-processing step. Wrapper based methods embed the model hypothesis search within the feature subset search. The performance of candidate feature subsets are evaluated [46]. The study reported in [46] gives a detailed explanation of the advantages and disadvantages of these methods: Filter based methods are fast, simple, scalable to high dimensional data. They handle the problem of finding a good feature subset for the classification process independently of the model selection step. On the other hand, wrapper methods consider the dependency between the features; feature and model search are performed interactively. These methods have higher risk of over fitting when compared to filter methods. they are computationally intensive, especially for high dimensional data; there is a critical need for devising heuristic strategies to search optimal feature subset. The embedded methods emerged as an alternative to the other types of methods in order to mediate their disadvantages. In these methods, the search for an optimal subset of features is incorporated in the classifier construction process, and hence can be seen as a search in the combined space of feature subsets and hypotheses.

In recent years, feature selection has become a prerequisite for most of the tasks that involve data analysis in bioinformatics [17]. Discarding the most irrelevant and redundant features and selecting the ones that are most relevant to the problem to be investigated helps in building robust learning models. Feature selection is principally used for dimensionality reduction, but besides it is also beneficial for enhancing the run-time of algorithms, for improving learning accuracy, and for enabling better model interpretability [18]. On the other hand, away from the other dimensionality reduction techniques, feature selection methods do not disrupt the specificity of variables by preserving their original semantics [17]. For the HIV-1 protease cleavage site prediction problem, the low sample count and high dimensionality can lead to over fitting [12], [19]. In this case, some attributes in spite of being unrelated to the target function can partition the samples very well [20]. Thus, applying feature selection to HIV-1 data appears as a crucial process to eliminate redundant features and to achieve the target of dimensionality reduction. In this context, we have proposed a consistency and SVM-based feature selection method for HIV-1 data in order to increase classification accuracy results. Other feature selection methods proposed for the HIV-1 protease cleavage site prediction problem can be found in [8], [12], [14]; and a comparison of the feature extraction methods is available in [13]. Additionally, good reviews on this subject can be found in [17]–[19]. Finally, one alternative to the consistency based feature selection method is the entropy based relevant and non-redundant feature selection method described in [39] where features are obtained for each class. However, in our method, the entire data set is taken into account for feature extraction.

## Methods

### Dataset

The input data is formed of octapeptide sequences and a class attribute which indicates whether the corresponding peptide is cleaved by HIV-1 protease or not. Each octapeptide sequence is composed of eight amino acids, denoted 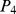, 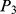, 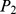, 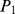, 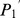, 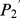, 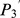, 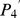, where each 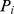 stands for one of the twenty possible amino acids. An example of an octapeptide which is found in the actual dataset is “AEELAEIF 1”, where the value ‘1’ denotes that this amino acid sequence is cleaved by HIV-1 protease. Additionally, cleaved sequences have a scissile bond located in the middle of an octapeptide sequence, namely between 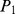 and 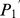 [2]. There are 20 different amino acids for each column and the data consists of 8 columns. Each method finds a different subset of features and at the end of the tests, FS-MLP and CFS-SVM are the methods that stand out. The dataset has the property that columns in the middle are more discriminative. When we analyze the HIV-1 dataset, the two columns at both ends are not very discriminative to predict cleavage and non-cleavage sites. On the other hand, columns in the middle can predict more decisively. This also has been observed in FS-MLP. The reason is that the scissile bond is in the middle. There are only few training and test datasets that can be accessed publicly for the HIV-1 protease cleavage site detection. This shortage of publicly available datasets constitutes an obstacle for generating solutions that can effectively tackle the problem. In 1998, Cai and Chou [6] used an expanded dataset with 362 peptides with 114 cleaved and 248 uncleaved samples for their neural network based technique to investigate cleave sites. Their work is a reiteration of the work by Thompson et al. [9]. This dataset has been used in several works, e.g., the works described in [22], [28]. Afterwards, another dataset with 392 new samples of which 281 cleaved and 111 uncleaved sequences were collected by Kim et al. [4]. Rule based approaches increase the interpretability of prediction. Their drawback is the number of rules to pick up for decision and overfitting. Finally, Oliviera et al. [29] published a dataset with 131 instances which are entirely cleaved octamers that do not exist in the datasets of Cai et al. or Kim et al. Neural network and MLP based approaches suffer from determining the number of hidden layers, determining the neurons to use in each hidden layer, and getting stuck at local minima. Convergence to an optimal solution is time consuming.

In the early publications on this topic, the first 362 instances dataset was used. After the collection of the second dataset, researchers used a combination of both, as 754 instances dataset with a total of 396 cleaved and 358 uncleaved sequences, without duplications. After the 131 instances dataset became publicly available, it has been used in several experiments as a test set while the 754 instances dataset is considered as the training set. We have used the same splitting in our work described in this paper. In recent works, the above mentioned three datasets are taken together into a final dataset leading to a dataset with 885 samples. These datasets are publicly available at the website http://www.cise.ufl.edu/~suchen/sbl [21]. On the other hand, we have noticed some conflict related to the dataset posted at this website, where it is stated that the 754 instances dataset contains 395 cleaved and 359 uncleaved samples, and a 133 instances dataset is available. However, in fact the 754 instances dataset consists of 396 cleaved and 358 uncleaved samples and the other dataset should be named 131-dataset as it comprises 131 samples.

**Figure 1:**
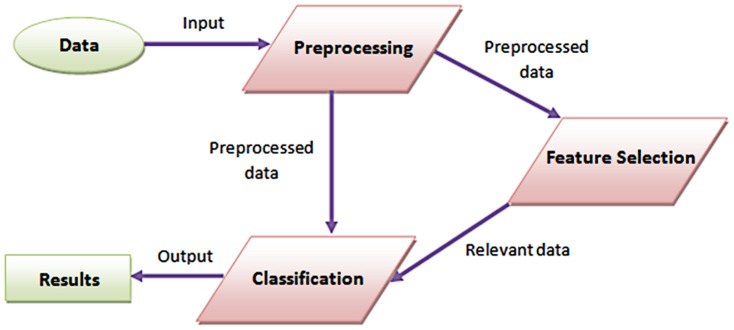
Overall System Architecture. The input data is preprocessed then the preprocessed data may be directly classified or feature selection is applied to utilize in the classification only relevant features.

The HIV-1 protease substrate sets consist of the genetically coded amino acids. For our purpose, we have ordered the amino acids as *G*, *A*, *P*, *V*, *L*, *I*, *M*, *F*, *Y*, *W*, *S*, *T*, *C*, *N*, *Q*, *K*, *H*, *R*, *D*, and *E*. Each of these amino acids can be located in 8 different indices. Thus, the feature space is composed of 160 attributes which can be denoted as <*G*_1_, *A*_1_, …, *E*_1_, …, *G*_8_, *A*_8_, …, *E*_8_>. In this notation, each *X_i_* stands for one of the amino acids with its index value. One of 20 amino acids with the same index *i* value is set to one, and the rest are set to zero. Our problem herein is to select the attributes most relevant to the data with the aim to increase classification accuracy results. To this end, we have proposed a hybrid feature selection method based on consistency and SVM-RFE (Recursive Feature Elimination). Within this system, the following steps are involved in sequence: preprocessing data, feature selection, and finally classification. The proposed system architecture is depicted in Figure 1.

**Figure 2:**
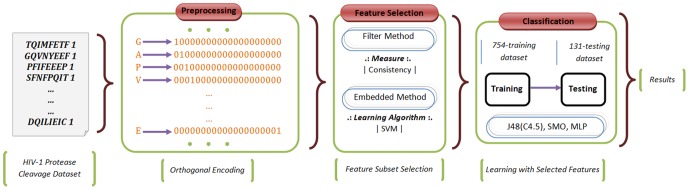
Detailed System Overview. Closer look at the various components of the proposed system architecture; orthonormal encoding is used to represent amino acids.

Before handling the data, as it is the case with many data mining techniques, a preprocessing operation is needed to clean the data. Next, in order to make a comparison within the system we have followed two distinct paths. One is directly classifying the preprocessed data and the other is classifying the data after the feature selection process as shown in Figure 1. Our aim is to emphasize the functionality and effectiveness of the feature selection task. Additionally, we have included the detailed system overview in Figure 2. In the following subsections, we present the steps applied prior to the classification process.

### Pre-processing data

In most data mining applications, it is required to filter the data before it is used in the latter phases of the data mining technique to be applied on the data. For our work, preliminary HIV-1 datasets were in categorical form where instances are represented by labels assigned to amino acids and the class label they belong to. However, the involved feature selection process stipulates a different representation of the data. Accordingly, we have applied a filtering operation on the preliminary dataset. In order to realize the feature selection process, the data should be represented by means of attributes. Thus, instances are encoded as binary values. For our example, each amino acid is represented by a 20-bit length string where each bit corresponds to an amino acid. For example, amino acid *G* is represented as 10000000000000000000, while amino acid *E* is represented as 00000000000000000001(see Figure 2), and the other amino acids in between *G* and *E* are represented as one bit shifted to the right at a time. This technique is also called orthonormal encoding. By representing an amino acid as 20-bit length string and by considering that a sequence comprises 8 indices, the total length of an instance increases to 160. Hereby, our objective is to select the best attributes from an array of 160 elements. This is the standard way of representing categorical data in terms of binary attributes [12].

### Feature selection

In this paper, we utilize these notations: *N* stands for the total number of attributes, which are 160 attributes for the case tackled in this paper; *F* represents the total number of selected features, and *I* is the total number of instances (754). The objective of the feature selection task is to find the optimal number of features within the given data. The most basic approach for this can lead to evaluating 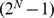 candidate subsets separately and selecting the best subset according to a measure criterion. However, finding the best feature subset would be an exhaustive iterative search in a feature space of size 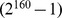. That makes it computationally infeasible. Accordingly, more realistic approaches are defined to evaluate subsets in a feature space. However, the evaluation of subsets is relative to the used function. Different evaluation functions can output different result subsets. According to the works described in [33], [34], evaluation functions are divided into five categories: information, distance, dependence, consistency, and classifier error rate.

Information measures try to determine the information gain provided by attributes. Distance measures select the attributes that ensure highest class severability. Dependence measures look for the correlation between features and classes. Consistency measures search for subsets that meet a certain inconsistency rate. Finally, classifier error rate measures use classifiers as measure functions to determine optimal feature subset within a feature space. Consistency measures are defined by an inconsistency rate. To calculate this rate, inconsistent and inconsistency count terms are defined additionally. Consistency measures consider two instances inconsistent if all their attributes match while their class labels are different. In other words, in a consistent set there are no two instances that have the same attributes and belong to different classes. For example, assume 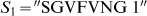 and 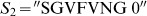 are two instances in a feature subset *S*. They match all but class labels. Thus, this pattern *p* is considered inconsistent due to having at least two instances pertaining to the defined situation.

Another term defined for consistency measure is inconsistency count. For instance, in the feature subset *S*, there are instances with the same attributes in addition to *S*_1_ and *S*_2_ whose total number is *N_p_*. Suppose that, from the latter instances *C*_1_ belongs to class 1 and *C*_2_ belongs to class 0; 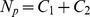, if 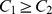, then the inconsistency count is computed as 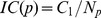, else it is computed as 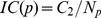. For the two classes example, this count ranges between 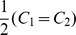 and 
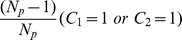
. Additionally, it is worth noting that the sum of patterns in a feature subset is equal to the total number of instances in the dataset 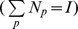. Finally, the inconsistency rate of a feature subset *S* is given by the sum of all patterns inconsistency counts in the feature subset divided by the instance count 
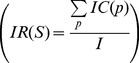
. For feature selection, this rate is used as follows: If a feature subset *S* has an inconsistency rate *IR*(*S*) below a predefined threshold value 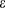, 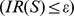 then subset *S* is considered to be consistent. In order to explore the space of consistent attribute subsets, a search method must be used.

According to the work described in [33], five different search techniques can be used to evaluate and select attributes from subsets for the consistency; these are exhaustive, complete, heuristic, probabilistic, and hybrid search methods. Exhaustive search time complexity is computationally infeasible, especially when the relevant feature count is high. Thus, generally more efficient techniques are preferred instead of the exhaustive search. Complete search starts with full set of features and continues its process by removing one feature at a time. It can be actually considered as a restricted version of exhaustive search; it reduces the time complexity. In heuristic search, at each iteration the remaining features are considered for selection or rejection. By probabilistic search, probabilistic choices are made to reach an optimal subset. And, hybrid search as the name implies uses a mixture of the aforementioned search strategies.

For our work, we have used consistency with a probabilistic search as implemented in the software that we have used. Probabilistic Las Vegas Filter algorithm (LVF) [41] has been adopted for the search procedure in which the inconsistency rate is used as the evaluation function. Whenever a consistent set is encountered (a set whose inconsistency rate is below a threshold value 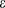 which is generally set to the inconsistency rate of the original set) its size is added to the size of the subset where it belongs. Since the inconsistency rate is monotonic, subsets with higher sizes are not evaluated any more.

Monotonicity of consistency can be defined as follows. Suppose that we have a number of subsets 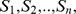 such that 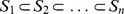. Thus, 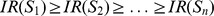. This characteristic of the consistency is a distinguishing feature which the other evaluation functions do not have. Owing to monotonicity, LVF proceeds fast when reducing the number of features as it ensures continuously lessening consistent subsets where supersets of the consistent sets are also consistent. Although, consistency is fast, noise-free, and good at removing irrelevant or redundant features, it is not obvious whether it can optimize the accuracy of the classifier that will be applied after the feature selection process, as it is the case with the other filter methods [35]. Thus, we have utilized an embedded method together with the consistency filter approach.

The work described in [22] shows that the HIV-1 dataset is linearly separable, and eventually using linear classifiers or extracting rules from linear models is as good as nonlinear approaches on the dataset. As linear models are so fast, we have decided to use a linear model to realize the feature selection task. Therefore, we have applied the SVM method of Recursive Feature Elimination (RFE); it is a linear SVM method that was proposed in [23]. This allows us to select the features most relevant to data.

SVM is able to handle a small amount of training data with a large number of features. SVM can be used when the data has two classes, and it determines the class for a given input [24]. To achieve this classification, SVM constructs a set of hyperplanes in a high-dimensional space and classifies data by finding the optimal hyperplane separating data points with maximal margin. An illustration of the described process is depicted in [23].

Suppose a set of points forming the training data is given, where 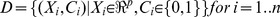. Each 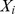 represents a real valued p-dimensional vector and 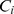 is either 0 or 1, indicating class label which implies cleaved or uncleaved samples for our example. SVM finds the hyperplane with maximum margin separating points that have 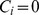 on one side and 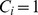 on the other side. A hyperplane can be represented by the formula 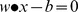, where *w* is the vector normal to the hyperplane, 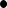 is the dot product, and *b* is the offset value. According to this equation, the value 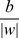 gives the distance between two groups (the distance between 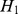 and 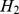, which is also called margin) and to maximize this distance, the two hyperplanes 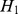 and 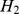 are described by the equations 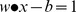 and 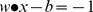. Thus, the distance between these hyperplanes becomes 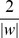; accordingly SVM tries to minimize 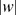. In some cases data may not enable a hyperplane for a straight separation. The techniques of soft margins and the kernel tricks are used to create a classifier to non-linear data. A review of the SVM methods and their optimization can be found in [25].

In this context, the SVM RFE algorithm ranks features according to their relevance to data by eliminating one feature at each iteration by default. In the algorithm, at the beginning SVM is employed on the training data. Then, the features are ordered by using the weights (weight vector *w*) of the classifier. Afterwards, the feature which has the smallest weight is eliminated. Thereafter, the process is repeated on the set restricted to the remaining features, and finally an order of ranked features is returned [26], [27].

### Classification techniques used for protease cleavage site prediction

Several classification techniques have been proposed for handling the HIV-1 cleavage problem. These techniques are based on machine learning methods like artificial neural networks (ANNs) [6], [7], [9], [28], support vector machines (SVMs) [10], [12], [22], [30], [36], and Decision Trees (DTs) [20], [21], [28]. They build classifiers capable of showing more complex relationships and interactions in a dataset than what traditional frequency-based and statistical methods can do [6], [10], [16].

For our work, we have applied in the experiments the three classification techniques that have been reported to have successful results over traditional methods [6], [10], [16]. Our C-FS-SVM is a combined method which contains column-consistency and column-SVM methods. We have also applied Consistency-SVM to show the results of combining consistency and column-SVM.

In addition to all these techniques, the work described in [12] uses MLP for prediction based on feature subset selection. In link to our subset selection method, we preferred to compare our method with the method described in [12] which uses MLP for classification (the feature subset selection methods described in [12] were reported to have outperformed existing feature selection methods). At the same time, we did not ignore the other two classification methods (SVM and Decision Trees). So, we have conducted experiments and comparative analysis by considering the three methods.

### Algorithm

In this section, we describe the details of the proposed algorithm which we have applied to select the most relevant features for the training data.

### Algorithm C-FS-SVM (training data, attribute number)

1. Rank the features by invoking the SVM-RFE algorithm on the whole feature set (on binary data) and point features in the range between 1 and feature count according to their relevance; then calculate column-based mean and standard deviation measures and store in set *A* features which have larger values than the sum of these measures for each column.
2. For each index (column) in the dataset, convert nominal data to orthogonal form and perform consistency-based attribute selection; then store the selected features in set *B*.
3. Compute the intersection of *A* and *B*, (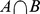) and get the mutual attributes located in each index, i.e., if any column returns null, then do not select any attribute related to the index, else select attributes that exist at the same indices in both sets.

**Figure 3:**
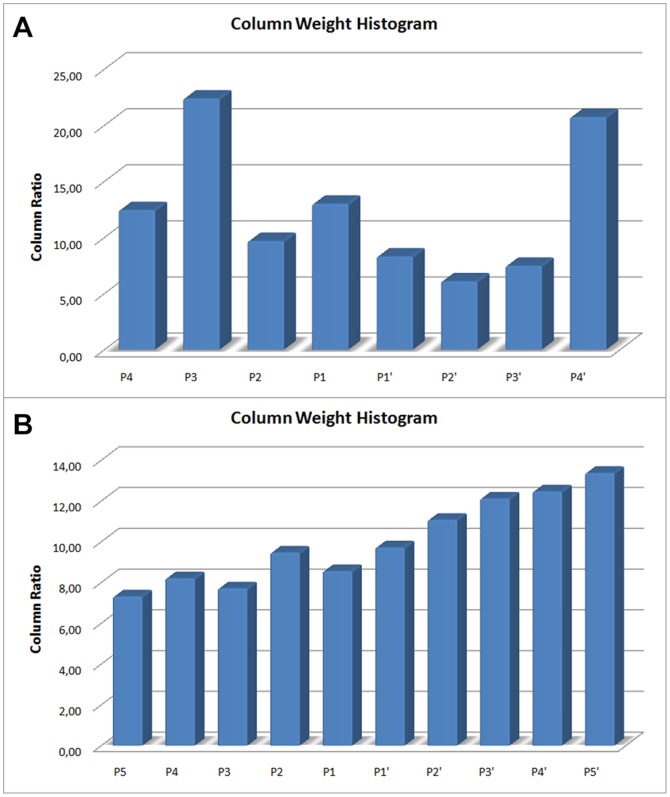
a) Nursery Data b) T-cell Data. Column weights obtained from SVM rank values (a), and (b). These are the total weight percentages of attributes (positional weight matrix) per index obtained as a result of SVM ranking.

**Figure 4:**
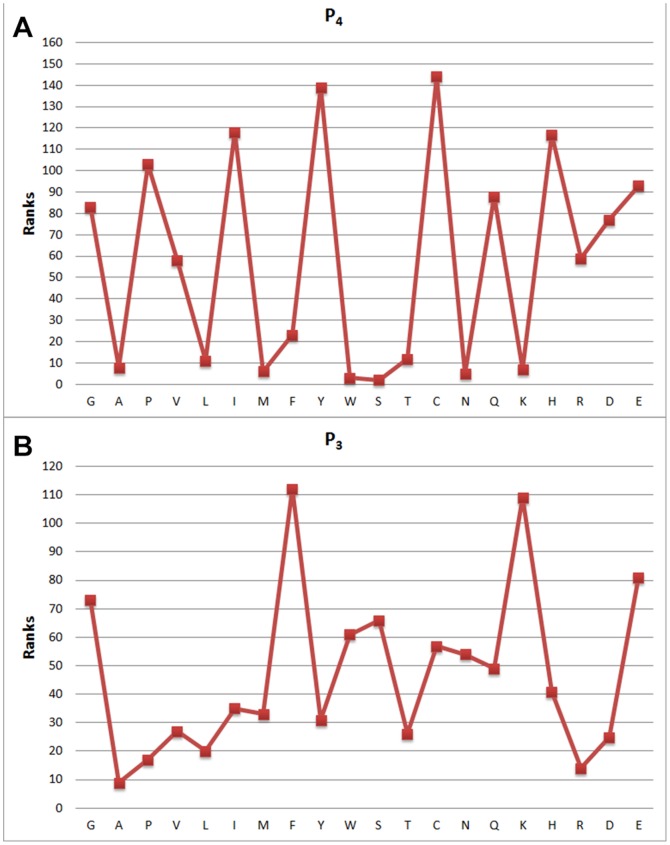
Feature weights of (a) *P*_4_ and (b) and *P*_3_.

**Figure 5:**
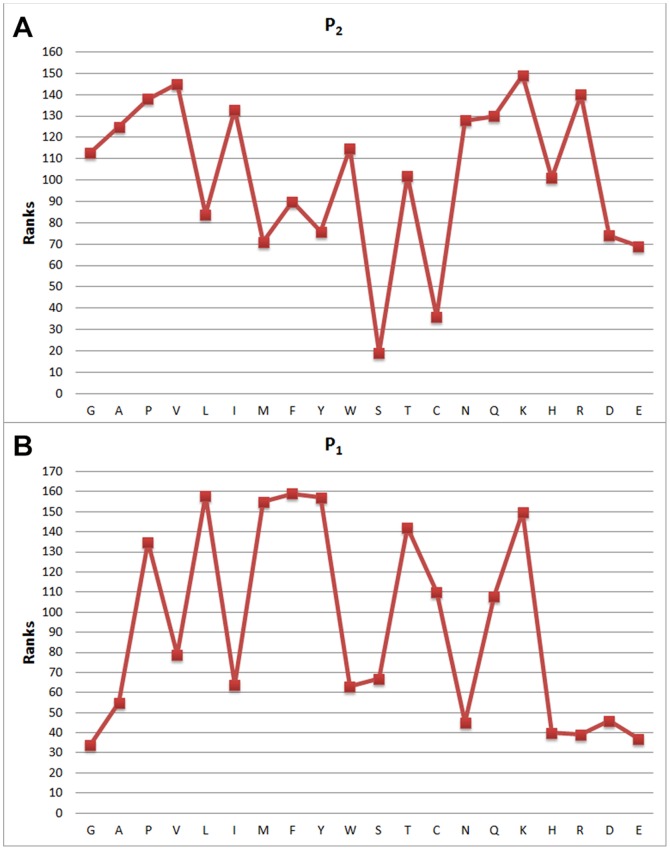
Feature weights of (a) 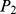 and (b) 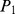 attribute values with respect to indices and the distribution of attribute weights.

**Figure 6:**
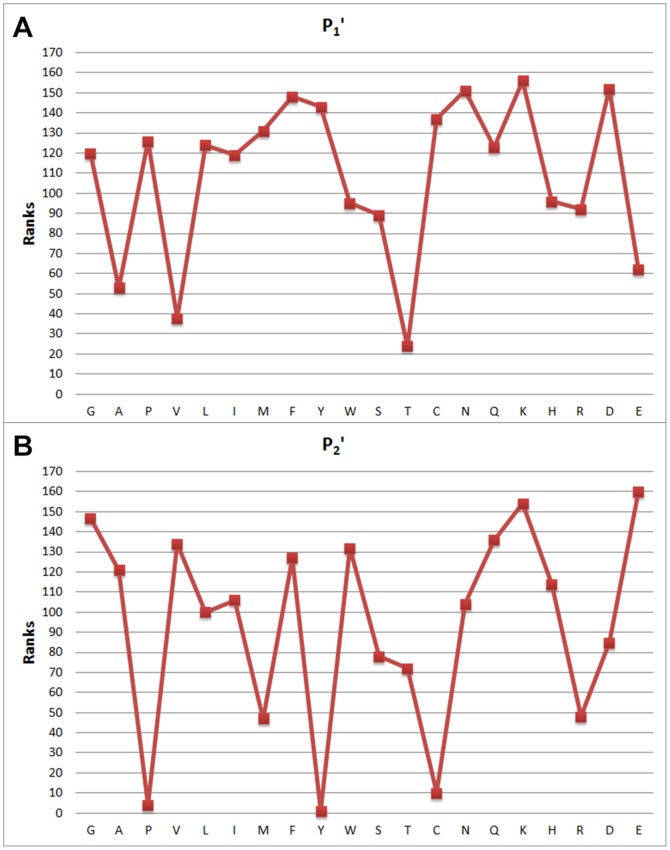
Feature weights of (a) *P*_1_' and (b) *P*_2_': attribute values with respect to indices and the distribution of attribute weights.

Figure 3, Figure 4, Figure 5 and Figure 6 show attribute values with respect to indices, and the distribution of attribute weights for each column is depicted. Especially the 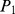 column shows that attributes within this column are much more conspicuous compared to the other ones as it has more cases passing the defined threshold value. According to this, we have concluded that columns show different tendencies and they should be evaluated separately. In this context, consistency-based evaluation of attributes has been made index-based rather than applied on the whole feature set.

The most recent results of the FS-MLP method presented by Kim et al. [12] render better performance compared to information gain, Relief, FS-SVM, and FS-P. Yet we have proposed C-FS-SVM involving column-consistency and column-SVM methods with three different classification techniques that are effective in HIV-1 protease cleavage site prediction [6], [10], [16] in order to measure their performance. We also proposed another method called consistency-SVM which is the combination of consistency and column-SVM. This method takes the intersection of features obtained after consistency and column-SVM separately.

Given in Table 1 are the features selected as a result of the index-based consistency (column-consistency), SVM-RFE with the defined threshold value (column-SVM), consistency evaluated on the full feature set (consistency), and our proposed algorithm (consistency-based feature selection with SVM, C-FS-SVM). We have also listed features selected by the intersection of consistency and column-SVM methods (consistency-SVM) which can be obtained by using consistency evaluation instead of column-based consistency method. All the selected features indicate that amino acids in the middle of the sequences are more distinctive than the ones closer to the endpoints. Additionally, notice that we have not selected any attributes in the columns where the intersection returns a null set. This is because a null set indicates that no attribute in this column shows a significant difference and no attribute within this column can be labeled as redundant or informative feature, i.e., its specificity cannot be determined. For FS-MLP we have used the stated attributes in the corresponding work and we have applied the selected features on the original feature set as we did for the other methods.

**Table 1:** Selected attributes according to the FS methods (- indicates no feature is specifically determined for the column).

FS Method	Num	P4 (@1)	P3 (@2)	P2 (@3)	P1 (@4)	P1' (@5)	P2' (@6)	P3' (@7)	P4' (@8)
**C-FS-SVM**	12	-	-	V	*F/L/M/Y*	D/*K*/N	G/*K*	*M*	*T*
**Column-Consistency**	37	P/S	C/S/T/V	N/V	*F/L/M/Y*	C/D/G/*K*/N/R/S/Q/T	C/D/F/G/H/*K*/M/N/S/W/Y	D/*M*/Q	P/*T*
**Column-SVM**	28	C/H/I/Y	E/F/K	K/R/V	*F*/K/*L*/*M*/T/*Y*	D/*K*/N	E/G/*K*	F/*M*/P	K/*T*/Y
**Consistency**	40	P/S/V/Q	F/G/S/T	F/G/K/P/Q/R/V/W	*F*/K/*L*/*M*/N/*Y*/V	D/F/G/*K*/L/N/R/S/Q	E/F/*K*/N/V/W	*M*/Q	-
**Consistency-SVM**	15	-	F	K/R/V	*F*/K/*L*/*M*/*Y*	D/*K*/N	E/*K*	*M*	-
**FS-MLP**	14	-	-	I/K/N/Q	*F*/K/*L*/*M*/*Y*	F/*K*/S	E/*K*	-	-

Values common to all methods have been italicized.

**Figure 7:**
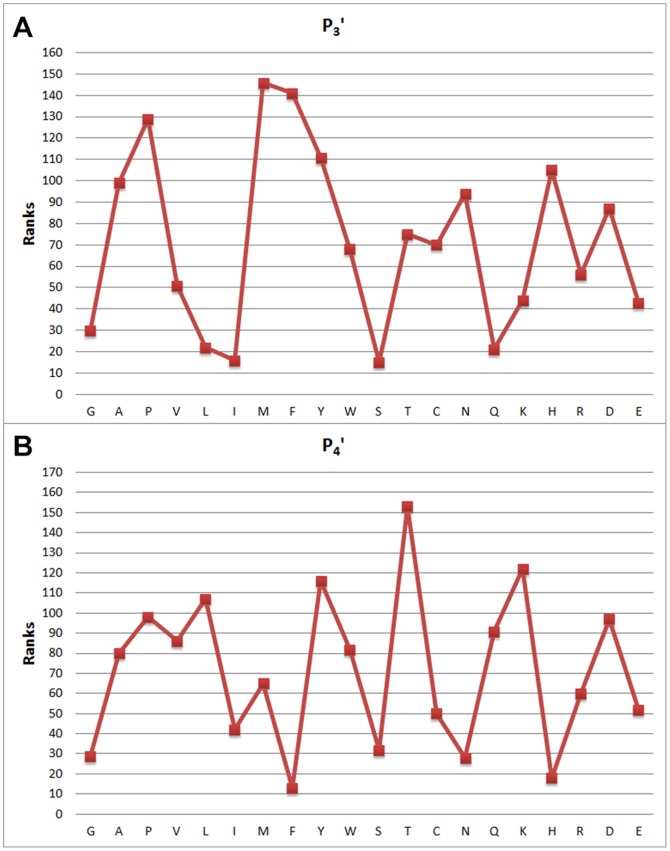
Feature weights of (a) *P*_3_' and (b) and *P*_4_': attribute values with respect to indices and the distribution of attribute weights.

The proposed C-FS-SVM algorithm consists of three main steps which are based on the consistency measure and the standard SVM-RFE algorithm. First, SVM-based feature selection is applied on the whole feature set and the most valuable/informative ones are selected according to a generic statistical method which is given by the sum of the mean (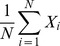) and the standard deviation 
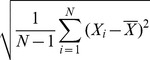
 of the values. Later, it determines features by separately evaluating the consistency-based measure on columns of the data. Finally, attributes located in both sets are returned. We evaluated the results of the SVM RFE algorithm for the Nursery [37] and the T-cell [38] datasets. Shown in Figure 7 are the total weight percentages of the attributes (positional weight matrix) per index obtained as a result of the SVM ranking. The distributions indicate that attributes in the nursery data are more correlated among themselves (the ones that are in the same column) while attributes in the T-cell data show more generic relationships. Evaluation of the proposed algorithm on the HIV-1 dataset is given in the following subsection.

## Experiments & Results

We have implemented our framework by using Weka [31], which is a data mining tool that has been extensively used for machine learning tasks. In Weka, preprocessing (filtering), clustering, classification, attribute selection, and association rules techniques can be realized with various options. In addition to the association rules mining and clustering, we have employed the other features that Weka provides. Filtering has been adapted to produce binary values as it was necessary to fulfill the attribute selection task. For attribute selection, consistency (Consistency Subset Evaluation) and SVM RFE (SVM Attribute Evaluation) have been used while for classification J48 Decision Tree, SMO (Support Vector Machines using Sequential Minimal Optimization), and MLP (Multilayer Perceptron) implementations of Weka have been used. Comprehensive testing has been done and the obtained results are given in the following subsections.

### Evaluation

In our experiments, we have used the entire dataset consisting of the 131-dataset [29] and the 46 uncleaved instances that have been selected from the 754-dataset in two level external cross validation fashion [42], [43]. As an alternative approach, we have used the 131-dataset for validation [29] in appendix 1 in File S1.

**Figure 8:**
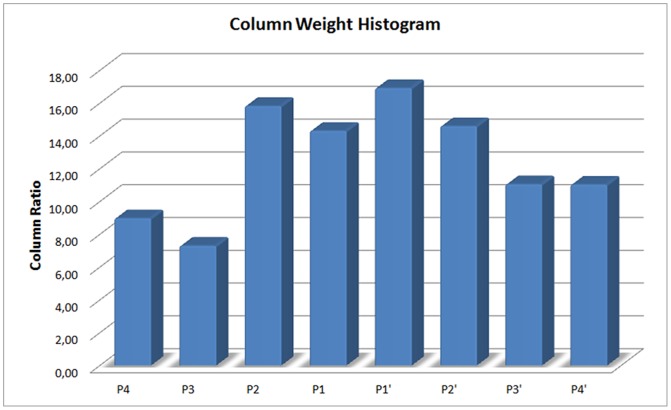
Column weights obtained from SVM rank values. These are the total weight percentages of attributes (positional weight matrix) per index obtained as a result of SVM ranking.

Shown in Figure 8 are the total weight percentages of the attributes (positional weight matrix) per index obtained as a result of SVM ranking. The results shown in Figure 8 indicate that attributes located in 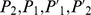 are much more distinctive than the ones located in 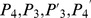. This is due to the fact that cleavage occurs between indices 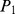 and 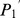; attributes appearing close to the scissile bond play more important role on the cleavage as already stated in [12].

While the ranker methods are good at ordering the relevance of attributes, it is important to know how to determine which of the ranked features should be selected, i.e., a suitable threshold value should be set in order to choose the top features. By following this type of methodology, while globally selecting top ranked features, we have also investigated the properties locally by examining the attributes within their corresponding indices by considering intra-relatedness. It can be inferred that the top 28 ranked features obtained via SVM RFE are not the same as the 28 features of our selection. Additionally, notice that our algorithm has not specified any attribute from the two columns 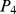 (@1) and 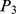 (@2); these columns showed the smallest total attribute weights in Figure 8. On the other hand, while column 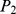 (@3) has the second most total attribute weight, only one attribute is specified for that column. This is because the specified attribute had a distinctive weight among attributes within the same column. Most attributes were selected for the two columns 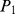 (@4) and 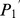 (@5), where the scissile bond takes place.

#### Evaluation with 5-fold external cross validation

The entire dataset with 885 instances has been used for two level external cross validation. The results point out that average classification results of SMO, MLP, and J48 have been used for comparing the No Feature Selection, the C-FS-SVM, the CFS, and the Relief methods. These experiments have been conducted 10 times for five-fold external cross-validation. Table 2, Table 3, Table 4 contain the standard deviations of the results in them including average performance results for accuracy, TP, FP, precision, recall, f-measure and ROC. All detailed accuracy, TP, FP, precision, recall, f-measure and ROC results have been given in Tables S11, S12, S13 in File S1.

**Table 2:** Standard Deviations of classification results for external cross validation with SMO and their average performance results for each metric.

Case	Method	Accuracy	TP	FP	Precision	Recall	FMeasure	ROC
Avg.	No Feature	87.89	0.82	0.10	0.76	0.82	0.79	0.86
	C-FS-SVM	**91.90**	**0.88**	**0.07**	**0.83**	**0.88**	**0.85**	**0.91**
	Relief	88.99	**0.88**	0.10	0.77	**0.88**	0.81	0.89
	CFS	88.38	0.85	0.10	0.76	0.85	0.80	0.87
Std. Dev.	No Feature	3.25	0.03	0.05	0.07	0.03	0.04	0.02
	C-FS-SVM	2.66	0.04	0.03	0.08	0.04	0.05	0.03
	Relief	4.47	0.03	0.06	0.08	0.03	0.05	0.03
	CFS	2.99	0.02	0.04	0.06	0.02	0.04	0.02

**Table 3:** Standard Deviations of classification results for external cross validation with MLP and their average performance results for each metric.

Case	Method	Accuracy	TP	FP	Precision	Recall	FMeasure	ROC
Avg.	No Feature	87.18	0.84	0.12	0.74	0.84	0.78	0.94
	C-FS-SVM	**88.82**	**0.87**	**0.11**	**0.77**	**0.87**	**0.81**	**0.95**
	Relief	86.75	0.88	0.14	0.73	0.88	0.79	0.94
	CFS	86.92	0.86	0.13	0.73	0.86	0.78	0.94
Std. Dev.	No Feature	3.62	0.03	0.05	0.07	0.03	0.04	0.02
	C-FS-SVM	5.29	0.04	0.07	0.11	0.04	0.07	0.02
	Relief	5.57	0.02	0.08	0.09	0.02	0.06	0.02
	CFS	3.55	0.02	0.05	0.06	0.02	0.04	0.02

**Table 4:** Standard Deviations of classification results for external cross validation with J48 and their average performance results for each metric.

Case	Method	Accuracy	TP	FP	Precision	Recall	FMeasure	ROC
Avg.	No Feature	87.17	0.74	**0.08**	**0.78**	0.74	0.75	0.86
	C-FS-SVM	**87.39**	**0.75**	0.09	**0.78**	**0.75**	**0.76**	0.85
	Relief	85.52	0.71	0.10	0.75	0.71	0.73	**0.87**
	CFS	86.48	0.73	0.09	0.77	0.73	0.74	0.86
Std. Dev.	No Feature	1.83	0.05	0.02	0.03	0.05	0.03	0.03
	C-FS-SVM	4.91	0.12	0.08	0.11	0.12	0.08	0.06
	Relief	3.14	0.07	0.04	0.05	0.07	0.05	0.03
	CFS	3.76	0.04	0.05	0.07	0.04	0.04	0.03

### Statistical significance of results

Wilcoxon signed rank test have been applied on the results of SMO, MLP and J48 presented in Appendix 4 in File S1. Table S14, Table S20, and Table S26 in File S1 for f-measure; and Table S15, Table S21, and Table S27 in File S1 for accuracy. As reported in Tables S14–S31 in File S1, our C-FS-SVM method outperforms the other methods. Results have been taken for different *α* levels (.90 and .95). In Wilcoxon tests, 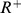 gives positive differences; 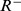 gives negative differences of C-FS-SVM against the no feature selection, the Relief, and the CFS methods.

The work described in [44], [45] suggests using Friedman Aligned test when the number of algorithms is low (4 or 5). Our C-FS-SVM method is held as the control algorithm and compared with multiple sign test based on a family of hypotheses (other algorithms), namely Holm-Hochberg-Hommel, Holland Finner, Li post hoc procedures. Again, all the results have been obtained for SMO, MLP, and J48 separately.

#### SMO

Average ranks obtained by each method in the Friedman Aligned test [44], [45] are reported in Table S32 in File S1. According to Table S32 in File S1, aligned Friedman statistic for f-measure (distributed according to chi-square with 3 degrees of freedom)is 7.689114. The p-value computed by Aligned Friedman Test for f-measure is 0.052893320524. Aligned Friedman statistic for accuracy (distributed according to chi-square with 3 degrees of freedom) is 7.755534. P-value computed by Aligned Friedman Test for accuracy is 0.051343724664. The p-values obtained by applying post hoc methods over the f-measure and accuracy results of Friedman Aligned procedure are reported in Tables S34 and S35 in File S1.

Different post-hoc procedures have been applied on fried aligned (Table S34 in File S1). According to P-values obtained in by applying post hoc methods over the accuracy results of Friedman Aligned procedure, Bonferroni-Dunn's procedure rejects hypotheses that have a p-value 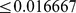. Holm's procedure rejects hypotheses that have a p-value 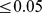. Hochberg's procedure rejects hypotheses that have a p-value 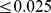. Hommel's procedure rejects hypotheses that have a p-value 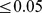. Holland's procedure rejects hypotheses that have a p-value 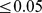. Rom's procedure rejects hypotheses that have a p-value 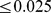. Finner's procedure rejects hypotheses that have a p-value 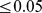. Li's procedure rejects hypotheses that have a p-value 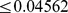.

Different post-hoc procedures have been applied on fried aligned (Table S35 in File S1). According to P-values obtained in by applying post hoc methods over the accuracy results of Friedman Aligned procedure, Bonferroni-Dunn's procedure rejects those hypotheses that have a p-value 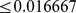. Holm's procedure rejects those hypotheses that have a p-value 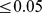. Hochberg's procedure rejects those hypotheses that have a p-value 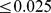. Hommel's procedure rejects those hypotheses that have a p-value 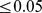. Holland's procedure rejects those hypotheses that have a p-value 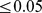. Rom's procedure rejects those hypotheses that have a p-value 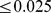. Finner's procedure rejects those hypotheses that have a p-value 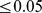. Li's procedure rejects those hypotheses that have a p-value 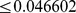.

#### MLP

Average ranks obtained by each method in the Friedman Aligned test [44], [45] are reported in Table S36 in File S1. According to Table S36 in File S1, aligned Friedman statistic for f-measure (distributed according to chi-square with 3 degrees of freedom) is 7.989851. The p-value computed by Aligned Friedman Test for f-measure is 0.046221926022. Aligned Friedman statistic for accuracy (distributed according to chi-square with 3 degrees of freedom) is 7.990249 and p-value computed by Aligned Friedman Test for accuracy: 0.046213661791. The p-values obtained by applying post hoc methods over the f-measure and accuracy results of Friedman Aligned procedure are reported in Tables S37 and S38 in File S1.

Different post-hoc procedures have been applied on fried aligned (Table S37 in File S1). According to P-values obtained in by applying post hoc methods over the accuracy results of Friedman Aligned procedure, Bonferroni-Dunn's procedure rejects hypotheses that have a p-value 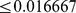. Holm's procedure rejects hypotheses that have a p-value 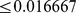. Hommel's procedure rejects hypotheses that have a p-value 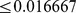. Holland's procedure rejects hypotheses that have a p-value 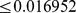. Finner's procedure rejects hypotheses that have a p-value 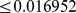. Li's procedure rejects hypotheses that have a p-value 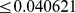. The p-values obtained by applying post hoc methods over the results of Friedman Aligned procedure are reported in Table S37 in File S1.

Different post-hoc procedures have been applied on fried aligned (Table S38 in File S1). According to P-values obtained in by applying post hoc methods over the accuracy results of Friedman Aligned procedure, Bonferroni-Dunn's procedure rejects those hypotheses that have a p-value 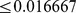. Holm's procedure rejects those hypotheses that have a p-value 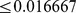. Hommel's procedure rejects those hypotheses that have a p-value 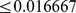. Holland's procedure rejects those hypotheses that have a p-value 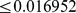. Finner's procedure rejects those hypotheses that have a p-value 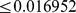. Li's procedure rejects those hypotheses that have a p-value 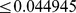. The p-values obtained by applying post hoc methods over the results of Friedman Aligned procedure are reported in Table S38 in File S1.

#### J48

Average ranks obtained by each method in the Friedman Aligned test [44], [45] are reported in Table S39 in File S1. According to Table S39 in File S1, Aligned Friedman statistic for f-measure(distributed according to chi-square with 3 degrees of freedom) is 8.078216. The p-value computed by Aligned Friedman Test for f-measure is 0.044422566866. Aligned Friedman statistic for accuracy(distributed according to chi-square with 3 degrees of freedom) is 8.071096. P-value computed by Aligned Friedman Test for accuracy is 0.044564988862. The p-values obtained by applying post hoc methods over the f-measure and accuracy results of Friedman Aligned procedure are reported in Tables S40 and S41 in File S1.

Different post-hoc procedures have been applied on fried aligned (Table S40 in File S1). According to P-values obtained in by applying post hoc methods over the accuracy results of Friedman Aligned procedure, Bonferroni-Dunn's procedure rejects hypotheses that have a p-value 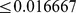. Holm's procedure rejects hypotheses that have a p-value 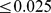. Hochberg's procedure rejects hypotheses that have a p-value 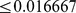. Hommel's procedure rejects hypotheses that have a p-value 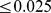. Holland's procedure rejects hypotheses that have a p-value 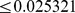. Rom's procedure rejects hypotheses that have a p-value 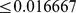. Finner's procedure rejects hypotheses that have a p-value 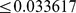. Li's procedure rejects hypotheses that have a p-value 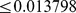.

Different post-hoc procedures have been applied on fried aligned (Table S41 in File S1). According to P-values obtained in by applying post hoc methods over the accuracy results of Friedman Aligned procedure, Bonferroni-Dunn's procedure rejects those hypotheses that have a p-value 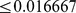. Holm's procedure rejects those hypotheses that have a p-value 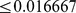. Hommel's procedure rejects those hypotheses that have a p-value 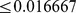. Holland's procedure rejects those hypotheses that have a p-value 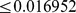. Finner's procedure rejects those hypotheses that have a p-value 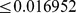. Li's procedure rejects those hypotheses that have a p-value 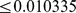.

## Conclusions

Curse of dimensionality is a crucial challenge for real life data. In order to handle this high dimensionality problem, feature selection or reduction techniques are extensively used as preprocessing step for data mining and knowledge discovery techniques. Data characterized by large number of features and low number of samples is difficult to be modeled by classifiers. Before or within the classification process, a feature selection operation is needed to be employed on this kind of data in order to help in developing effective classifiers. HIV-1 protease cleavage site prediction is an interesting classification problem and the data related to this problem also requires preprocessing by considering the feature selection phase. To realize this phase, we have proposed a hybrid approach which is capable of selecting the best features describing the data. We have compared our results with state-of-art and generic methods which have been applied on HIV-1 data. The reported results indicate that our method is capable of selecting features that improve classification results significantly. It has been shown that the selected features have different impacts on different classifiers. The proposed C-FS-SVM method is based on consistency measure and the SVM RFE algorithm which examines the supplied data in two distinct forms, and then combines the results obtained from each form. The performance of C-FS-SVM has showed that evaluating the features in the data separately and combining the outcome with a global selection is prone to give more accurate results. Additionally, determining a threshold value is essential for the feature selection problem because it is mostly not possible to know the optimal number of features that could represent the data or at least a precise number of features is not given in advance. For this, we have developed a reliable selection criterion where we are selecting the most distinctive attributes from both global and local perspectives. In conclusion, we have proposed a hybrid method for selecting features related to HIV-1 data. By utilizing the selected features, we acquired considerable enhancements over the classification results. As a future work, we are planning to add physiochemical properties that amino acids have and we will investigate encoding schemes based on our method.

## Supporting Information

File S1 **Supplementary Evaluation and Tables.**
(PDF)
