# Supplementary material for: A Consistency-Based Feature Selection Method Allied with Linear SVMs for HIV-1 Protease Cleavage Site Prediction
Source: PLoS One. 2013 Aug 23;8(8):e63145. doi: 10.1371/journal.pone.0063145 (PMC3751940; doi:10.1371/journal.pone.0063145)
Supplement: File S1 — Supplementary Evaluation and Tables. (PDF) [file pone.0063145.s001.pdf]

1 APPENDIX A: Evaluation with 131-dataset studied in [4, 8, 12]

As a first step in the algorithm, the attributes have been assigned scores from 160 (total attribute count in the HIV-1 data) to 1 according to the ranked values returned by SVM-RFE. Then, the average value of each attribute has been taken into account column by column. We have used 10-fold cross validation of data while selecting attributes.

The confusion matrices are given reported Tables 24-30 in Appendix 2. Class labels are given as a and b. The value b indicates that the HIV-1 Protease Cleavage Site has been found (scissile bond exists). Vertical labels are the actual classes and horizontal labels are the predicted classes.

We have analyzed results in two parts. The first part considers feature subset selection (C-FS-SVM, Column-Consistency, Column-SVM, Consistency, Consistency-SVM, FS-MLP, Sequence Logo, Relief, FS-P, FS-SVM, InfoGain, PCA, PLS) also including no feature selection in Table S1. Here, they have the classification results for J48, SMO, and MLP. The second part covers reported results of methods with 160 features(PWM, Assoc. Rules, C4.5 rules, C4.5 + boosting rules, NN/GSVM, NN rules w/blackbox, NN rules w/decomp.) and they are compared against C-FS-SVM in Table S2.

Results given in Table S1 contains the precision(= 1), sensitivity(equal to accuracy since all instances are positive), f-measure results. We used reported feature subsets in [12] and reran the classifiers in order to validate the scores and gave the results of consistency, FS-MLP, and No feature selection for two cases: 1) J48, MLP, SMO Classification with current attribute values 2) current attribute values in orthonormal encoding(results were as reported in [12]). These results(no feature, consistency, FS-MLP) have been reported in pairs respectively and delimited with "/". According to the results reported in Table S1, our feature selection approach has enhanced classification results (except C4.5 decision tree) and provides the highest accuracies for SVM and MLP classifiers for all the methods. We have previously mentioned that the original feature space size is 20^8 and to perform feature selection orthonormal encoding is used which makes the domain space sparser by increasing it from 20^8 to 2^{160}. However, we have represented the selected features in their original domain space.

Table S1 summarizes the precision, sensitivity, accuracy, and the f-measure classification results. The 131-dataset contains only the positive classes and accordingly we have obtained the value 1 for precision from all the methods. Accuracy score is the same as sensitivity score. However, recall values differ between methods and so do the f-measure values.

For the J48 classification, FS-MLP outperforms the other methods in the decision tree with 95.42% and 97.66% for the recall and the f-measure, respectively. The No Feature Selection, Column-Consistency,

Column-SVM methods have 93.13% and 96.44% results for the recall and the f-measure, respectively. No Feature Selection results remain the same for SMO and MLP as well. C-FS-SVM and Consistency methods perform slightly worse with the recall and the f-measure reported as 92.37% and 96.03%, respectively. The consistency-SVM method had 91.60% and 95.62% for the recall and the f-measure, respectively. However, the results reported by our proposed methods are very close to the results of FS-MLP for the J48 classification.

For sequential minimization optimization and the MLP classifiers, our methods are better by far. FS-MLP reported 88.55%/94.65% and 93.93%/97.25% results for the recall and the f-measure whereas the No Feature selection method reported 93.13%/93.12% and 96.44%/96.43% for the two measures. The Consistency method has 93.13%/87.02% and 96.44%/93.05% for the recall and the f-measure (for SMO). We obtained 90.84% and 95.20% for the recall and the f-measure which are slightly better than the measures of FS-MLP with the MLP classifier when classification is performed with current data. However, the situation changes when orthonormal encoding in classification is used, Column-SVM produced better results than the consistency method in SMO against FS-MLP and Consistency methods(96.95% and 98.45% for the recall and the f-measure). The second best method is the Column-Consistency method. It returned 96.95% and 98.45% for the recall and the f-measure (similar to Column-SVM in SMO) and 95.42% and 97.66% for the recall and the f-measure (MLP). C-FS-SVM is superior to all the other methods. It reported the recall and the f-measure values as 97.71% and 98.84% for SMO and 98.47% and 99.23% for MLP.

Sensitivity and accuracy results are the same and specificity results are all undefined since the validation dataset contains only positive instances. This is reported in Table S1. FS-MLP achieved 95.42%/95.41% for J48, and it is slightly better than the Column-Consistency, the Column-SVM, and the No Feature Selection methods. C-FS-SVM reported 92.37%. Consistency-SVM reported 92.37%/90.83%. The worst result is 91.60% in Consistency-SVM. It can be seen that although FS-MLP has better results than all the others, the results are comparative to each other for J48. C-FS-SVM is significantly better than FS-MLP for SMO (97.71%) and MLP (98.47%) by far. No feature selection remains the same at 93.13%/90.83% for J48; 93.13%/93.12% for SMO; and 93.13%/91.60% for MLP.Column-Consistency and Column-SVM produced the second best result for SMO (96.95%). Column-Consistency is still the second in MLP with a slight decrease (95.42%). Consistency-SVM sensitivity score (96.18%) was better than that of Consistency and FS-MLP for both results, which are 93.13%/87.02%(Consistency) and 88.55%/94.65%(Consistency)for SMO. Consistency-SVM is better than Consistency and and FS-MLP when the first values in pairs at FS-MLP and Consistency are are considered. FS-MLP is better when their second values are taken into account.

We have also given the collection of results reported in [4] at Table S2. Accuracy and sensitivity results

are the same. Precision is 1 since all are positive instances and specificity results are not available because the dataset contains only positive class instances. NN rules w/decomp. Method [4], C4.5 + boosting rules [4], NN/GSVM classifier [4],NN rules w/blackbox methods [4] are remarkable in Table S2. However, they don't have feature selection stage. NN rules w/decomp. obtained the same score as in CFS-SVM with SMO but C-FS-SVM with MLP is better with 12 selected features.

Suppose that the full feature set is defined as AA, the space of the selected features is denoted SE, and the space of the unselected features is denoted SE'. Considering $SE = V@3\vee$ $[L/M/F/Y]@4\vee$ $[N/K/D]@5\vee$ $[G/K]@6\vee$ $M@7\vee$ $T@8$, SE' would be $\neg SE$, where features are the ones that do not appear in SE and are connected with $\wedge$. Then, we can determine the size of the selected feature space by computing $|SE| = |AA| - |SE'|$ $(AA = SE \cup SE')$, where $|AA|$ is 2.56×10^{10} $(\approx 20^8)$, and $|SE'| \approx 1.34 * 10^{10}(\approx 20 \times 20 \times 19 \times 16 \times 17 \times 18 \times 19 \times 19)$; eventually $|SE| \approx 1.22 * 10^{10}$. This way the feature space is reduced by half. However, by considering sparser feature space where $|AA| = 2^{160}$ and $|SE| \approx 1.22 * 10^{10} < 2^{34}$, it can be seen that the size of the feature space is greatly reduced. On the other hand, selecting features from their original space allows the data to preserve its originality and the election of samples in the training data. For all the examples, we have employed classifiers on the original (nominal) data where each attribute is represented by a label. Here it is worth mentioning that encoding is a distinct issue in the HIV-1 protease problem which is generally made by using amino acids' physicochemical properties. An encoding technique offered for the specified problem and some general information can be found in [32]. Additionally, for the indicated classifiers we have used Weka's default parameters while taking hidden layer number 6 (h=6) for the MLP classifier for all the examples.

Statistical Significance of Results In order to evaluate the statistical significance of the obtained results, we conducted a permutation test on the entire dataset which includes 754 training and 131 test instances. The f-measure scores were used in the experiments. Since SMO and MLP are two competitive methods for feature selection, p-values based on the these classifiers (SMO and MLP) were calculated and are reported in Table S3. There are four columns in Table S3; these columns stand for: no feature selection with SMO, our method (C-FS-SVM) with SMO, no feature selection with MLP, our method (C-FS-SVM) with MLP, respectively. The values reported in Table S3 reflect ten repetitive experiments. For each set, the 885-dataset has been split into 66% for training and 34% for testing. Then, the obtained scores were used to calculate the p-values.

For SMO, the two-tailed p-value is 0.0028 (t=4.0702, df=9), and for MLP the two-tailed p-value is 0.0026 (t=4.0702, df=9). By conventional criteria, the difference is considered to be statistically significant.

Table S1 Precision(Pr.),Sensitivity(Recall or Rec.), and F-Measure results according to the 131-dataset; it is worth noting that this dataset contains only positive classes and hence the precision value is reported as '1' and accuracy and sensitivity scores are the same

FS Method(# of selected features)	J48			SMO			MLP		
	Pr.	Rec.	F-Measure	Pr.	Rec.	F-Measure	Pr.	Rec.	F-Measure
No Feature S.(160)	1	93.13/90.83	96.44/95.19	1	93.13/93.12	96.44/96.43	1	93.13/91.60	96.44/95.61
C-FS-SVM(12)	1	92.37	96.03	1	97.71	98.84	1	98.47	99.23
Column-Consistency(37)	1	93.13	96.44	1	96.95	98.45	1	95.42	97.66
Column-SVM(28)	1	93.13	96.44	1	96.95	98.45	1	89.31	94.35
Consistency(40) [8,12]	1	92.37/90.83	96.03/95.19	1	93.13/87.02	96.44/93.05	1	90.84/85.49	95.20/92.17
Consistency-SVM(15)	1	91.60	95.62	1	96.18	98.05	1	93.89	96.85
FS-MLP w/decay [8,12](14 or 21)	1	95.42/95.41	97.66/97.65	1	88.55/94.65	93.93/97.25	1	88.55/94.65	93.93/97.25
Sequence Logo [8,12](9)	1	33.58	50.276988	1	33.58	50.28	1	35.87	52.80
InfoGain [8](21)	1	58.77	74.03	1	62.59	76.99	1	58.77	74.03
InfoGain [12](14)	1	58.77	74.03	1	62.59	76.99	1	58.77	74.03
Relief [12](14)	1	58.77	74.03	1	58.01	73.43	1	58.77	74.03
Relief [8](21)	1	58.77	74.03	1	56.48	72.19	1	61.83	76.41
FS-P [8](21)	1	90.82	95.19	1	91.09	95.34	1	92.15	95.91
FS-P [12](14)	1	87.78	93.49	1	91.60	95.62	1	60.30	75.23
FS-SVM [8](21)	1	89.31	94.35	1	84.73	91.73	1	90.07	94.78
FS-SVM [12](14)	1	90.83	95.19	1	91.60	95.62	1	83.20	90.83
PCA [12](126)	1	58.77	74.03	1	96.94	98.45	1	95.41	97.65
PLS [12](8)	1	60.30	75.23	1	58.01	73.43	1	63.35	73.43

Table S2 Other Classification Results Reported in [4]

FS Method	No of Features	Accuracy	Precision	Sensitivity	F-Measure
PWM rules [4]	160	25.95	1	25.95	41.21
Assoc. Rules [4]	160	27.48	1	27.48	43.11
C4.5 rules [4]	160	74.04	1	74.04	85.08
C4.5 + boosting rules [4]	160	93.89	1	93.89	96.85
NN/GSVM classifier [4]	160	90.84	1	90.84	95.20
NN rules w/blackbox Method [4]	160	90.08	1	90.08	94.78
NN rules w/decomp. Method [4]	160	97.71	1	97.71	98.84

Table S3 F-measure score of no feature selection vs SMO and MLP to be used for hypothesis testing with p-values

set#	SMO		MLP(h=a)	
1	0.893	0.902	0.884	0.899
2	0.93	0.933	0.93	0.933
3	0.888	0.898	0.895	0.899
4	0.903	0.906	0.893	0.899
5	0.917	0.919	0.914	0.927
6	0.89	0.913	0.893	0.912
7	0.92	0.933	0.913	0.92
8	0.913	0.919	0.91	0.933
9	0.904	0.917	0.898	0.901
10	0.917	0.92	0.904	0.907

2 APPENDIX B

Table S4 Confusion Matrix of No Feature Selection

	Confusion Matrix		
**Classifier**	**a**	**b**	
**J48**	0	0	**a=0**
	9	122	**b=1**
**SMO**	0	0	**a=0**
	9	122	**b=1**
**MLP**	0	0	**a=0**
	9	122	**b=1**

Table S5 Confusion Matrix of C-FS-SVM

	Confusion Matrix		
**Classifier**	**a**	**b**	
**J48**	0	0	**a=0**
	10	121	**b=1**
**SMO**	0	0	**a=0**
	3	128	**b=1**
**MLP**	0	0	**a=0**
	2	129	**b=1**

Table S6 Confusion Matrix of Column-Consistency

	Confusion Matrix		
**Classifier**	**a**	**b**	
**J48**	0	0	**a=0**
	9	122	**b=1**
**SMO**	0	0	**a=0**
	4	127	**b=1**
**MLP**	0	0	**a=0**
	6	125	**b=1**

Table S7 Confusion Matrix of Column-SVM

	Confusion Matrix		
**Classifier**	**a**	**b**	
**J48**	0	0	**a=0**
	9	122	**b=1**
**SMO**	0	0	**a=0**
	4	127	**b=1**
**MLP**	0	0	**a=0**
	14	117	**b=1**

Table S8 Confusion Matrix of Consistency

	Confusion Matrix		
**Classifier**	**a**	**b**	
**J48**	0	0	**a=0**
	10	121	**b=1**
**SMO**	0	0	**a=0**
	9	122	**b=1**
**MLP**	0	0	**a=0**
	12	119	**b=1**

Table S9 Confusion Matrix of Consistency-SVM

	Confusion Matrix		
**Classifier**	**a**	**b**	
**J48**	0	0	**a=0**
	11	120	**b=1**
**SMO**	0	0	**a=0**
	5	126	**b=1**
**MLP**	0	0	**a=0**
	8	123	**b=1**

Table S10 Confusion Matrix of FS-MLP

	Confusion Matrix		
**Classifier**	**a**	**b**	
**J48**	0	0	**a=0**
	6	125	**b=1**
**SMO**	0	0	**a=0**
	15	116	**b=1**
**MLP**	0	0	**a=0**
	15	116	**b=1**

3 APPENDIX C

Table S11 Classification Results for external cross validation with SMO

Case	Method	Accuracy	TP	FP	Precision	Recall	FMeasure	ROC
1	No Feature	88.72	0.77	0.07	0.8	0.77	0.78	0.85
	C-FS-SVM	92.67	0.83	0.04	0.88	0.83	0.85	0.89
	Relief	90.48	0.85	0.08	0.8	0.85	0.82	0.89
	CFS	88.71	0.82	0.09	0.76	0.82	0.79	0.87
2	No Feature	87.38	0.86	0.12	0.73	0.86	0.79	0.87
	C-FS-SVM	97.74	0.93	0.01	0.98	0.93	0.96	0.96
	Relief	90.89	0.88	0.08	0.8	0.88	0.84	0.9
	CFS	88.86	0.86	0.1	0.76	0.86	0.81	0.88
3	No Feature	85.86	0.83	0.13	0.71	0.83	0.76	0.85
	C-FS-SVM	89.83	0.87	0.09	0.77	0.87	0.82	0.89
	Relief	86.72	0.89	0.14	0.7	0.89	0.78	0.87
	CFS	85.81	0.85	0.14	0.7	0.85	0.76	0.86
4	No Feature	89.64	0.84	0.08	0.79	0.84	0.81	0.88
	C-FS-SVM	93.23	0.91	0.06	0.84	0.91	0.88	0.93
	Relief	91.89	0.89	0.07	0.82	0.89	0.85	0.91
	CFS	91.14	0.86	0.07	0.81	0.86	0.84	0.9
5	No Feature	89.77	0.79	0.06	0.82	0.79	0.8	0.86
	C-FS-SVM	89.25	0.85	0.09	0.76	0.85	0.8	0.88
	Relief	91.43	0.82	0.05	0.85	0.82	0.83	0.88
	CFS	89.79	0.81	0.07	0.81	0.81	0.81	0.87
6	No Feature	89.31	0.83	0.09	0.78	0.83	0.8	0.87
	C-FS-SVM	93.34	0.85	0.04	0.89	0.85	0.87	0.91
	Relief	89.49	0.87	0.09	0.77	0.87	0.81	0.89
	CFS	89.77	0.82	0.08	0.8	0.82	0.81	0.87
7	No Feature	79.49	0.84	0.22	0.59	0.84	0.69	0.81
	C-FS-SVM	91.53	0.96	0.1	0.77	0.96	0.85	0.93
	Relief	77.05	0.89	0.27	0.56	0.89	0.68	0.81
	CFS	80.92	0.87	0.21	0.61	0.87	0.71	0.83
8	No Feature	88.52	0.79	0.08	0.78	0.79	0.78	0.85
	C-FS-SVM	90.15	0.85	0.08	0.79	0.85	0.82	0.89
	Relief	90.22	0.86	0.08	0.79	0.86	0.82	0.89
	CFS	88.5	0.84	0.1	0.75	0.84	0.79	0.87
9	No Feature	89.42	0.84	0.09	0.78	0.84	0.81	0.88
	C-FS-SVM	92.6	0.85	0.05	0.86	0.85	0.86	0.9
	Relief	89.67	0.91	0.11	0.75	0.91	0.82	0.9
	CFS	90.11	0.87	0.09	0.78	0.87	0.82	0.89
10	No Feature	90.76	0.85	0.07	0.81	0.85	0.83	0.89
	C-FS-SVM	88.7	0.87	0.11	0.74	0.87	0.8	0.88
	Relief	92.04	0.9	0.07	0.82	0.9	0.86	0.91
	CFS	90.15	0.87	0.09	0.79	0.87	0.83	0.89
Avg.	No Feature	87.89	0.82	0.10	0.76	0.82	0.79	0.86
	C-FS-SVM	**91.90**	**0.88**	**0.07**	**0.83**	**0.88**	**0.85**	**0.91**
	Relief	88.99	**0.88**	0.10	0.77	**0.88**	0.81	0.89
	CFS	88.38	0.85	0.10	0.76	0.85	0.80	0.87

Table S12 Classification Results for external cross validation with MLP

	Method	Accuracy	TP	FP	Precision	Recall	FMeasure	ROC
1	No Feature	88.89	0.78	0.07	0.8	0.78	0.79	0.94
	C-FS-SVM	89.08	0.82	0.09	0.79	0.82	0.8	0.95
	Relief	89.82	0.84	0.08	0.79	0.84	0.81	0.96
	CFS	87.99	0.81	0.09	0.76	0.81	0.78	0.94
2	No Feature	85.82	0.87	0.15	0.7	0.87	0.77	0.93
	C-FS-SVM	95.38	0.93	0.04	0.9	0.93	0.91	0.97
	Relief	86.64	0.88	0.14	0.72	0.88	0.79	0.94
	CFS	85.58	0.87	0.15	0.69	0.87	0.77	0.92
3	No Feature	84.02	0.87	0.17	0.67	0.87	0.75	0.93
	C-FS-SVM	76.04	0.88	0.28	0.53	0.88	0.66	0.91
	Relief	82.58	0.88	0.19	0.64	0.88	0.73	0.93
	CFS	84.58	0.87	0.16	0.68	0.87	0.75	0.93
4	No Feature	88.77	0.85	0.1	0.77	0.85	0.8	0.94
	C-FS-SVM	91.2	0.85	0.07	0.82	0.85	0.84	0.96
	Relief	90.04	0.9	0.1	0.78	0.9	0.83	0.96
	CFS	90.78	0.87	0.08	0.8	0.87	0.83	0.96
5	No Feature	88.85	0.81	0.08	0.79	0.81	0.79	0.94
	C-FS-SVM	89.52	0.82	0.08	0.79	0.82	0.8	0.94
	Relief	89.67	0.86	0.09	0.79	0.86	0.82	0.95
	CFS	88	0.84	0.11	0.75	0.84	0.79	0.94
6	No Feature	89.2	0.85	0.09	0.77	0.85	0.81	0.95
	C-FS-SVM	91.91	0.86	0.06	0.85	0.86	0.85	0.97
	Relief	88.51	0.89	0.12	0.74	0.89	0.8	0.95
	CFS	89.32	0.83	0.08	0.78	0.83	0.8	0.95
7	No Feature	78.27	0.84	0.24	0.58	0.84	0.68	0.89
	C-FS-SVM	86.45	0.95	0.17	0.67	0.95	0.79	0.95
	Relief	72.27	0.88	0.33	0.51	0.88	0.64	0.88
	CFS	78.37	0.87	0.25	0.58	0.87	0.69	0.9
8	No Feature	89.14	0.82	0.08	0.79	0.82	0.8	0.94
	C-FS-SVM	90.44	0.89	0.09	0.78	0.89	0.83	0.96
	Relief	89.92	0.85	0.08	0.79	0.85	0.81	0.95
	CFS	88.77	0.86	0.1	0.75	0.86	0.8	0.95
9	No Feature	89.25	0.86	0.09	0.77	0.86	0.81	0.95
	C-FS-SVM	92.38	0.84	0.05	0.87	0.84	0.85	0.97
	Relief	88.97	0.91	0.12	0.74	0.91	0.81	0.96
	CFS	89.48	0.89	0.1	0.77	0.89	0.82	0.96
10	No Feature	89.62	0.86	0.09	0.78	0.86	0.82	0.96
	C-FS-SVM	85.83	0.88	0.15	0.68	0.88	0.77	0.95
	Relief	89.09	0.9	0.11	0.75	0.9	0.82	0.96
	CFS	86.35	0.87	0.14	0.72	0.87	0.78	0.95
Avg.	No Feature	87.18	0.84	0.12	0.74	0.84	0.78	0.94
	C-FS-SVM	**88.82**	**0.87**	**0.11**	**0.77**	**0.87**	**0.81**	**0.95**
	Relief	86.75	0.88	0.14	0.73	0.88	0.79	0.94
	CFS	86.92	0.86	0.13	0.73	0.86	0.78	0.94

Table S13 Classification Results for external cross validation with J48

Case	Method	Accuracy	TP	FP	Precision	Recall	FMeasure	ROC
1	No Feature	86.29	0.67	0.07	0.78	0.67	0.72	0.83
	C-FS-SVM	81.36	0.5	0.08	0.7	0.5	0.58	0.71
	Relief	84.09	0.58	0.07	0.75	0.58	0.65	0.89
	CFS	86.56	0.68	0.07	0.78	0.68	0.72	0.82
2	No Feature	89.18	0.83	0.08	0.79	0.83	0.8	0.89
	C-FS-SVM	89.83	0.72	0.04	0.87	0.72	0.79	0.82
	Relief	89.36	0.83	0.08	0.79	0.83	0.81	0.91
	CFS	85.2	0.79	0.13	0.71	0.79	0.75	0.86
3	No Feature	85.79	0.68	0.08	0.76	0.68	0.72	0.81
	C-FS-SVM	90.96	0.8	0.05	0.84	0.8	0.82	0.79
	Relief	85.6	0.64	0.07	0.77	0.64	0.7	0.81
	CFS	85.56	0.67	0.08	0.76	0.67	0.71	0.81
4	No Feature	88.57	0.76	0.07	0.8	0.76	0.78	0.87
	C-FS-SVM	89.27	0.8	0.08	0.79	0.8	0.8	0.89
	Relief	88.18	0.74	0.07	0.79	0.74	0.76	0.87
	CFS	89.19	0.75	0.06	0.82	0.75	0.78	0.88
5	No Feature	88.26	0.76	0.08	0.79	0.76	0.77	0.86
	C-FS-SVM	88.7	0.78	0.08	0.78	0.78	0.78	0.83
	Relief	87.51	0.77	0.09	0.77	0.77	0.77	0.87
	CFS	89.28	0.76	0.06	0.81	0.76	0.79	0.87
6	No Feature	88.48	0.75	0.07	0.81	0.75	0.77	0.87
	C-FS-SVM	89.83	0.78	0.06	0.82	0.78	0.8	0.86
	Relief	84.66	0.73	0.11	0.72	0.73	0.72	0.87
	CFS	88.19	0.75	0.07	0.79	0.75	0.76	0.86
7	No Feature	82.9	0.73	0.14	0.7	0.73	0.7	0.86
	C-FS-SVM	76.27	0.93	0.3	0.52	0.93	0.67	0.9
	Relief	77.98	0.72	0.2	0.63	0.72	0.66	0.83
	CFS	76.54	0.74	0.23	0.6	0.74	0.65	0.83
8	No Feature	87.51	0.7	0.06	0.81	0.7	0.75	0.84
	C-FS-SVM	86.29	0.66	0.07	0.78	0.66	0.71	0.84
	Relief	86.78	0.7	0.07	0.77	0.7	0.73	0.87
	CFS	87.73	0.72	0.07	0.79	0.72	0.75	0.87
9	No Feature	87.7	0.74	0.08	0.78	0.74	0.76	0.89
	C-FS-SVM	92.09	0.83	0.05	0.86	0.83	0.84	0.94
	Relief	86.52	0.72	0.08	0.76	0.72	0.74	0.88
	CFS	88.33	0.75	0.07	0.79	0.75	0.77	0.89
10	No Feature	87.03	0.73	0.08	0.77	0.73	0.75	0.87
	C-FS-SVM	89.27	0.7	0.04	0.86	0.7	0.77	0.87
	Relief	84.52	0.71	0.11	0.72	0.71	0.71	0.88
	CFS	88.23	0.71	0.06	0.81	0.71	0.76	0.9
Avg.	No Feature	87.17	0.74	**0.08**	**0.78**	0.74	0.75	0.86
	C-FS-SVM	**87.39**	**0.75**	0.09	**0.78**	**0.75**	**0.76**	0.85
	Relief	85.52	0.71	0.10	0.75	0.71	0.73	**0.87**
	CFS	86.48	0.73	0.09	0.77	0.73	0.74	0.86

4 APPENDIX D

Table S14 Results obtained by the Wilcoxon test for the C-FS-SVM algorithm versus the other three methods with SMO for f-measure

VS	R^+	R^-	Exact P-value	Asymptotic P-value
No Feature Selection	44.0	1.0	0.007812	0.005598
Relief	37.5	7.5	0.08593999999999999	0.058447
CFS	51.5	3.5	0.011719	0.006851

Table S15 Results obtained by the Wilcoxon test for algorithm C-FS-SVM versus the other three methods with SMO for accuracy

VS	R^+	R^-	Exact P-value	Asymptotic P-value
No Feature Selection	51.0	4.0	0.013672	0.014433
Relief	44.0	11.0	0.10546	0.083131
CFS	52.0	3.0	0.009766	0.010827

Table S16 Confidence intervals for the C-FS-SVM algorithm with SMO for f-measure(α=0.90)

α=0.90	Confidence interval	Exact confidence
No Feature Selection	[0.03 , 0.11]	0.91602
Relief	[0 , 0.08]	0.91602
CFS	[0.015 , 0.095]	0.91602

Table S17 Confidence intervals for algorithm C-FS-SVM with SMO for f-measure(α=0.95)

α=0.95	Confidence interval	Exact confidence
No Feature Selection	[0.02 , 0.115]	0.95118
Relief	[0 , 0.09]	0.95118
CFS	[0.015 , 0.1]	0.95118

Table S18 Confidence intervals for algorithm C-FS-SVM with SMO for accuracy(α=0.90)

α=0.90	Confidence interval	Exact confidence
Algorithm 1	[1.535 , 7.155]	0.91602
Algorithm 3	[0.005 , 5.57]	0.91602
Algorithm 4	[1.285 , 6.225]	0.91602

Table S19 Confidence intervals for algorithm C-FS-SVM with SMO for accuracy(α=0.95)

α=0.95	Confidence interval	Exact confidence
Algorithm 1	[0.985 , 7.195]	0.95118
Algorithm 3	[-0.115 , 6.85]	0.95118
Algorithm 4	[1.06 , 6.42]	0.95118

Table S20 Results obtained by the Wilcoxon test for the C-FS-SVM algorithm with MLP for f-measure

VS	R^+	R^-	Exact P-value	Asymptotic P-value
No Feature Selection	40.0	15.0	≥ 0.2	0.156998
Relief	35.5	19.5	≥ 0.2	0.367677
CFS	45.0	10.0	0.08398	0.053204

S21 Results obtained by the Wilcoxon test for algorithm C-FS-SVM with MLP for accuracy

VS	R^+	R^-	Exact P-value	Asymptotic P-value
No Feature Selection	40.0	15.0	≥ 0.2	0.185144
Relief	38.0	17.0	≥ 0.2	0.262193
CFS	44.0	11.0	0.10546	0.083131

Table S22 Confidence intervals for the C-FS-SVM algorithm with MLP for f-measure (α=0.90)

α=0.90	Confidence interval	Exact confidence
No Feature Selection	[-0.01 , 0.075]	0.91602
Relief	[-0.015 , 0.07]	0.91602
CFS	[0.005 , 0.065]	0.91602

Table S23 Confidence intervals for the C-FS-SVM algorithm with MLP for f-measure(α=0.95)

α=0.95	Confidence interval	Exact confidence
No Feature Selection	[-0.02 , 0.075]	0.95118
Relief	[-0.02 , 0.08]	0.95118
CFS	[0 , 0.075]	0.95118

Table S24 Confidence intervals for the C-FS-SVM algorithm with MLP for accuracy (α=0.90)

α=0.90	Confidence interval	Exact confidence
No Feature Selection	[-1.245 , 5.115]	0.91602
Relief	[-1.37 , 6.07]	0.91602
CFS	[0.285 , 5.11]	0.91602

Table S25 Confidence intervals for algorithm C-FS-SVM with MLP for accuracy (α=0.95)

α=0.95	Confidence interval	Exact confidence
No Feature Selection	[-1.8 , 5.43]	0.95118
Relief	[-1.57 , 6.72]	0.95118
CFS	[-0.23 , 5.445]	0.95118

Table S26 Results obtained by the Wilcoxon test for the C-FS-SVM algorithm with J48 with f-measure

VS	R^+	R^-	Exact P-value	Asymptotic P-value
No Feature Selection	31.5	23.5	≥ 0.2	0.637872
Relief	41.0	14.0	0.19336	0.143105
CFS	37.5	17.5	≥ 0.2	0.246517

Table S27 Results obtained by the Wilcoxon test for algorithm C-FS-SVM with J48 with accuracy

VS	R^+	R^-	Exact P-value	Asymptotic P-value
No Feature Selection	33.0	22.0	≥ 0.2	0.540818
Relief	42.0	13.0	0.16016	0.126279
CFS	36.0	19.0	≥ 0.2	0.358951

Table S28 Confidence intervals for the C-FS-SVM algorithm with J48 for f-measure(α=0.90)

α=0.90	Confidence interval	Exact confidence
No Feature Selection	[-0.03 , 0.045]	0.91602
Relief	[-0.005 , 0.07]	0.91602
CFS	[-0.025 ,0.05]	0.91602

Table S29 Confidence intervals for algorithm C-FS-SVM with J48 for f-measure(α=0.95)

α=0.95	Confidence interval	Exact confidence
No Feature Selection	[-0.04 , 0.05]	0.95118
Relief	[-0.015 , 0.08]	0.95118
CFS	[-0.04 ,0.055]	0.95118

Table S30 Confidence intervals for algorithm C-FS-SVM with J48 for accuracy(α=0.90)

α=0.90	Confidence interval	Exact confidence
No Feature Selection	[-2.195 , 2.545]	0.91602
Relief	[-0.31 , 3.33]	0.91602
CFS	[-0.72 , 2.74]	0.91602

Table S31 Confidence intervals for algorithm C-FS-SVM with J48 for accuracy(α=0.95)

α=0.95	Confidence interval	Exact confidence
No Feature Selection	[-2.64 , 2.87]	0.95118
Relief	[-0.62 , 4.96]	0.95118
CFS	[-1.01 , 3.135]	0.95118

Table S32 Average Rankings of the algorithms with SMO for f-measure and accuracy(Aligned Friedman)

Algorithm	Ranking(f-measure)	Ranking(accuracy)
No Feature Selection	30.55	28.8
C-FS-SVM	9.5	9.8
Relief	17.35	18.05
CFS	24.6	25.35

Table S34 Post Hoc comparison Table for $\alpha = 0.05$ with SMO f-measure(FRIEDMAN ALIGNED)

algorithm	$z = (R_0 - R_i)/SE$	p	Holm Hochberg Hommel	Holland	Rom	Finner	Li
No Feature S.	4.026297	0.000057	0.016667	0.016952	0.016667	0.016952	0.04562
CFS	2.888223	0.003874	0.025	0.025321	0.025	0.033617	0.04562
Relief	1.501493	0.133228	0.05	0.05	0.05	0.05	0.05

Table S35 Accuracy Post Hoc comparison Table for $\alpha = 0.05$ with SMO accuracy (FRIEDMAN ALIGNED)

algorithm	$z = (R_0 - R_i)/SE$	p	Holm Hochberg Hommel	Holland	Rom	Finner	Li
No Feature S.	3.634187	0.000279	0.016667	0.016952	0.016667	0.016952	0.046602
CFS	2.974295	0.002937	0.025	0.025321	0.025	0.033617	0.046602
Relief	1.578002	0.114565	0.05	0.05	0.05	0.05	0.05

Table S36 Average Rankings of the algorithms with MLP for f-measure and accuracy(Aligned Friedman)

Algorithm	Ranking(f-measure)	Ranking(accuracy)
No Feature Selection	23.4	22
C-FS-SVM	14.05	14
Relief	20.35	21.6
CFS	24.2	24.4

Table S37 Post Hoc comparison Table for $\alpha = 0.05$ with MLP f-measure (FRIEDMAN ALIGNED)

algorithm	$z = (R_0 - R_i)/SE$	p	Holm Hochberg Hommel	Holland	Rom	Finner	Li
CFS	1.941421	0.052207	0.016667	0.016952	0.016667	0.016952	0.040621
No Feature S.	1.788403	0.073711	0.025	0.025321	0.025	0.033617	0.040621
Relief	1.20502	0.228196	0.05	0.05	0.05	0.05	0.05

Table S38 Accuracy Post Hoc comparison Table for $\alpha = 0.05$ with MLP accuracy(FRIEDMAN ALIGNED)

algorithm	$z = (R_0 - R_i)/SE$	p	Holm Hochberg Hommel	Holland	Rom	Finner	Li
Algorithm 4	1.989239	0.046675	0.016667	0.016952	0.016667	0.016952	0.044945
Algorithm 1	1.530184	0.125971	0.025	0.025321	0.025	0.033617	0.044945
Algorithm 3	1.453675	0.146036	0.05	0.05	0.05	0.05	0.05

Table S39 Average Rankings of the algorithms with J48 for f-measure and accuracy(Aligned Friedman)

Algorithm	Ranking(f-measure)	Ranking(accuracy)
No Feature Selection	16.9	17.3
C-FS-SVM	15.15	16
Relief	29.1	28.15
CFS	20.85	20.55

Table S40 Post Hoc comparison Table for $\alpha = 0.05$ with J48 f-measure(FRIEDMAN ALIGNED)

algorithm	$z = (R_0 - R_i)/SE$	p	Holm Hochberg Hommel	Holland	Rom	Finner	Li
Relief	2.668259	0.007625	0.016667	0.016952	0.016667	0.016952	0.013798
CFS	1.090256	0.2756	0.025	0.025321	0.025	0.033617	0.013798
No Feature S.	0.334728	0.73783	0.05	0.05	0.05	0.05	0.05

Table S41 Accuracy Post Hoc comparison Table for $\alpha = 0.05$ with J48 accuracy(FRIEDMAN ALIGNED)

algorithm	$z = (R_0 - R_i)/SE$	p	Holm Hochberg Hommel	Holland	Rom	Finner	Li
Relief	2.323967	0.020127	0.016667	0.016952	0.016667	0.016952	0.010335
CFS	0.870292	0.384141	0.025	0.025321	0.025	0.033617	0.010335
No Feature S.	0.248655	0.803628	0.05	0.05	0.05	0.05	0.05
